# Global airways: a Danish nationwide real-life registry of biologic therapy for chronic rhinosinusitis with nasal polyps

**DOI:** 10.3389/falgy.2025.1735943

**Published:** 2026-01-13

**Authors:** Vibeke Backer, Kasper Aanæs, Bent Ivan Larsen, Christiane Haase, Anne-Sophie Homøe, Jens Tidemandsen, Therese Ovesen, Adnan Madzak, Grethe Samuelsen, Jonas Hjelm Andersen, Lars Christian Meyer, Kristian Bruun Petersen, Thorbjørn Hermanrud, Lars Peter Schousboe, Christian Korsgaard Pedersen, Kjeld Hansen, Søren Pauli, Mads Vrelits Filtenborg, Bibi Lange, Preben Homøe, Christian von Buchwald, Anette Kjeldsen

**Affiliations:** 1 Department of Otorhinolaryngology, Head & Neck Surgery, and Audiology, Rigshospitalet, Copenhagen University Hospital, Copenhagen, Denmark; 2 Department of Clinical Medicine, University of Copenhagen, Copenhagen, Denmark; 3 Department of Oto-rhino-laryngology, and Maxillofacial Surgery, Zeeland University Hospital, Køge, Denmark; 4 Department of Clinical Medicine, University of Southern Denmark, SDU, Odense, Denmark; 5 Department of Regional Health Research, University of Southern Denmark, SDU, Koege, Denmark; 6 Department of Oto-rhino-laryngology, Head and Neck Surgery, Gødstrup Hospital, Herning, Denmark; 7 Department of Clinical Medicine, Aarhus University, Aarhus, Denmark; 8 Department of Oto-rhino-laryngology, Head and Neck Surgery, Nordsjaellands Hospital, Hilleroed, Denmark; 9 Department of Oto-rhino-laryngology, Head and Neck Surgery, Esbjerg Grindsted, Esbjerg, Denmark; 10 Department of Oto-rhino-laryngology, Head and Neck Surgery, Lillebaelt Hospital, Vejle, Denmark; 11 Department of Oto-rhino-laryngology, Head and Neck Surgery, Aarhus University Hospital, Skejby, Denmark; 12 Center for Clinical Research and Prevention, Frederiksberg Hospital, Copenhagen, Denmark; 13 Department of Otorhinolaryngology, Head and Neck Surgery, Aalborg University Hospital, Aalborg, Denmark; 14 Department of Clinical Medicine, Aalborg University, Aalborg, Denmark; 15 Department of Otorhinolaryngology, Head & Neck Surgery, and Audiology, Odense University Hospital, Odense, Denmark

**Keywords:** asthma, chronic rhinosinusitis with nasal polyps, monoclonal antibodies, real-life data, registry, type 2 inflammation

## Abstract

**Background:**

The relationship between chronic rhinosinusitis (CRS) with nasal polyps (CRSwNP) and type 2 inflammation has led to the use of biologic treatment for uncontrolled cases. As biologic treatment remains a relatively new approach for CRSwNP, systematic assessment and collection of high-quality, real-world data are crucial. This study established the national Global Airways registry to collect longitudinal data over a period of 12 months for patients with CRSwNP treated with biological therapies according to criteria established by the Danish Health Authority.

**Methods:**

All participating sites conducted systematic assessments of patients with CRSwNP referred for initiation of biologic treatment. Clinical and patient-reported outcome data were collected at baseline and after 6 and 12 months of treatment and were entered in real-time into the Global Airways registry. Comparisons were performed between patients eligible or not eligible for biologic therapy and between pre- and post-treatment timepoints.

**Results:**

A total of 513 patients were enrolled between November 2022 and December 2024, with 310 receiving treatments with biologics (mepolizumab or dupilumab). Mean [standard deviation (SD)] age in the treatment group was 49.7 (14) years and 66% were male. The median number of previous endoscopic sinus surgeries was 2 (range 1–16). Baseline mean (SD) scores were as follows: Nasal Polyp Score (NPS) 4.8 (1.7); Sinonasal Outcome Test (SNOT)-22 68.7 (18.7); Visual Analog Scale (VAS) CRS 84.1 (16); and Sniffin’ Sticks-16 (SST-16) score 4.8 (3). Asthma was present in 204 (66%) patients, with a mean (SD) Asthma Control Questionnaire (ACQ)-5 score of 2.1 (1.5). Among patients with available data at both 6 and 12 months (*n* = 160), mean SNOT-22 scores improved from 68 to 29 and 24, NPS from 5.1 to 3.0 and 2.4, SST-16 from 4.7 to 9.2 and 10.0, and ACQ-5 from 2.3 to 1.0 and 0.8 (all *p* < 0.001).

**Conclusions:**

The Global Airways registry was an effective working tool that ensured collection of important real-world data when moving from surgery to biologics. Furthermore, the registry demonstrated the sustained effectiveness of biologic therapy in patients with refractory CRSwNP and provided a robust foundation for defining CRS phenotypes and advancing targeted treatment strategies.

## 1 Introduction

Chronic rhinosinusitis (CRS) is a persistent inflammatory condition of the nasal and paranasal mucosa requiring lifelong treatment. It is typically classified into two phenotypes: CRS with nasal polyps (CRSwNP) and CRS without nasal polyps (1). CRSwNP affects approximately 3%–4% of the Nordic population (2), and it is often associated with asthma and type 2 (T2) inflammation (3–5).

Patients with CRSwNP often report impaired quality of life (6, 7), high recurrence rates (8) and incomplete disease control. A higher T2 inflammatory burden, characterized by comorbid asthma and high eosinophilic counts, is associated with an increased risk of NP recurrence (4, 8, 9). Standard of care (SoC) includes topical steroids and nasal saline irrigation, often supplemented with courses of antibiotics and/or systemic corticosteroids (SCS), as well as endoscopic sinus surgery (ESS) (10–12).

Understanding the T2 inflammatory pathway has provided a rationale for treating with monoclonal antibodies (biological drugs; hereafter referred to as ‘biologics’) (13–17). Clinical trials of biologics have demonstrated significant reductions in both Nasal Polyp Scores (NPS; 1–2 point reduction) and Sinonasal Outcome Test (SNOT-22) scores [exceeding the minimal clinically important difference (MCID) of an 8.9 point reduction] (18–22). However, real-world studies have reported even greater improvements for both outcomes (23–25) and a recent large cohort study of almost 1,000 patients in Italy demonstrated remission rates of 42.7% at 12 months and 64.2% at 24 months with dupilumab (26). The true rate of complete on-treatment remission in real-world settings may vary across populations and treatment strategies, highlighting the need for robust real-world registries to account for differences in geography, ethnicity, and treatment possibilities.

In 2022, the Danish Medicines Council approved the use of biologics for patients with ongoing severe, uncontrolled CRSwNP despite adherence to SoC and previous ESS (27). Following this approval, all ear-nose-and throat (ENT) departments who initiated treatment with biologics for CRSwNP were required to register patient data in a nationwide registry (Global Airways). The aim of the present study is to report the design and implementation of the prerequisite Global Airways registry and to report baseline characteristics and clinical outcomes from the first year of biologic treatment in patients with severe CRSwNP and comorbidities.

## 2 Methods

### 2.1 Study design and inclusion criteria

The Global Airways registry included all patients referred to ENT departments for possible eligibility for biologic treatment from November 2022. Nine of ten ENT departments in Denmark applied for approval to treat with biologics and all patients were referred either from a private ENT, a secondary ENT department or a secondary asthma clinic. Treatment was initiated in patients with confirmed CRSwNP (28) who meet four mandatory criteria: 1) presence of bilateral NPS; 2) ESS under general anesthesia within the last three years (or contraindication to surgery); 3) signs of T2 inflammation; and 4) self-reported adherence to intranasal corticosteroids (INCS) >80%. Signs of T2 inflammation were defined as 1) high blood eosinophil count (defined as a current count ≥0.25 × 10^6^ /L or a history of ≥0.30 × 10^6^ /L); 2) moderate or severe tissue eosinophilic cell counts [10–100 or >100 per high-power field (hpf)]; 3) IgE >100 kU/L; or 4) FeNO >50 parts per billions (ppb). Adherence to INCS was assessed by the Medication Adherence Report Scale [MARS-5] and an in-house self-reported questionnaire. Furthermore, patients were required to fulfill at least three of five additional criteria: A) two courses of SCS (25–37.5 mg for 5–7 days, or 34 mg depot injection), or low dose SCS (5–10 mg) for one period of 3 months per year, or a contraindication to SCS (e.g., osteoporosis, diabetes); B) a SNOT-22 score ≥ 50; C) Sniffin’ Sticks 16 (SST-16) result ≤8; D) NPS ≥5; or E) asthma diagnosed and treated with inhaled corticosteroids (ICS).

Patients who met all four mandatory criteria (1–4) and at least three of the five additional criteria (A–E) were offered biologic therapy. Exceptions were granted for a few patients for medical reasons, including CRSwNP with concomitant Pott's puffy tumor or mucocele, contraindication to INCS due to central serous chorioretinopathy (CSCR), current successful treatment in a randomized controlled trial (RCT), inability to fill out questionnaires due to intellectual disability or documented anxiety preventing surgery. Patients who did not meet the eligibility criteria could be re-screened at future timepoints. Females planning pregnancy were advised to refrain from biologic treatment.

All patients gave informed consent to having their data included in the national database. The study was approved by the Danish Research Ethics Committee (H-21020685) and conducted under a data protection agreement (P-2021-193, Global Airways).

### 2.2 Treatment

Patients received either mepolizumab (100 mg every 4 weeks) or dupilumab (300 mg every 2 weeks for 6 months and subsequently every 4 weeks). Mepolizumab is the reimbursed first-choice biologic for CRSwNP in Denmark, but many patients received dupilumab as they were participating in an ongoing head-to-head randomized trial of mepolizumab vs. dupilumab (TORNADO; EU clinical trial ID 2022-50-22-50-14-00). Omalizumab is reimbursed in Denmark in case of pregnancy but has not yet been used in this study. To maintain the integrity of the ongoing TORNADO RCT, differences in outcomes between mepolizumab and dupilumab were not analyzed in this current study.

Continuation of treatment at six and 12 months is guided by Danish authority-defined response criteria: 1) reduction in NPS of ≥1 point at 6 months or ≥2 points at 12 months; 2) reduction in SNOT-22 score of ≥12 points; 3) change from anosmia (0–8) to normosmia (9–16) as measured by SST-16; and 4) reduction in Asthma Control Questionnaire (ACQ) of ≥0.5 points. Continuation of biologic therapy required patients to meet at least two out of these four criteria. Patients scoring below two points at six months may have continued treatment based on medical indication. Treatment was discontinued if a patient required rescue therapy, such as ESS or SCS.

### 2.3 Outcomes

Demographic and lifestyle data were collected for all patients, including sex, height, weight, and body mass index (BMI). Smoking status was recorded as current, never, or ex-smoker (≥6 months abstinent). Sinonasal examination included rhinoscopy performed by an ENT specialist, with NPS scored from 0 to 4 on each side (29). Current treatments for CRSwNP were documented, including adherence to INCS, use of nasal saline irrigation, and use of SCS. Olfactory function was evaluated using the SST-16 test, with scores ≤8 indicating anosmia and 9–16 indicating hyposmia/normosmia. NSAID-exacerbated respiratory disease (N-ERD) was recorded based on patient-reported symptoms, without provocation testing. All patients were evaluated for allergic rhinitis, defined as symptoms and a positive allergy test (30).

Bronchial assessment included specialist evaluation for asthma, defined by symptoms and a positive asthma challenge test (reversibility testing and bronchial challenge testing with mannitol or methacholine) (31). ICS were initiated or continued as clinically required. Spirometry [forced expiratory volume in 1 s [FEV_1_], forced vital capacity [FVC] and FEV_1_/FVC ratio] was performed with predicted values calculated in REDCap. Fractional exhaled nitric oxide (FeNO) measurements were performed by the ENT clinic. T2 inflammation was defined as described in the eligibility criteria.

All patients completed patient outcome questionnaires, including SNOT-22, ACQ-5/7, and visual analog scale (VAS) scores for CRS, asthma, smell function, and N-ERD (VAS scores ranged from 0 to 100, with 100 denoting the worst severity).

A full list of outcomes collected as part of the registry are provided in Supplementary Appendix A1.

### 2.4 Registry procedures

Each participating ENT department was responsible for the systematic assessment of both upper and lower airways. The registry included licensed use of the SNOT-22, Asthma Control Test (ACT), and ACQ instruments. Prior to the initiation of the registry, local meetings were held at all sites to train staff in Global Airways diseases, systematic assessments, local treatment with INCS and ICS, and the workflow of the registry, which has been developed as a clinical support tool.

Research staff assigned a unique identification number to each patient, including a suffix denoting the geographic region of assessment. Data could be entered in the Global Airways registry by trained airway nurses, by patients using a QR code, or by physicians. Data entry did not need to be completed in a single session, and examinations could be split across multiple visits, with dates recorded for each assessment. All data from the baseline evaluation was entered into the registry and the program generated a recommendation on whether to initiate biologic treatment.

All evaluations described above were repeated every 6 months. An additional shorter visit was conducted 4 months after initiation of biologic therapy with a focus on systemic or local side effects.

Full details of all registry procedures are provided in Supplementary Appendix A2.

### 2.5 Statistical analysis

All analyses were performed using SAS Enterprise Guide version 8.3. Standard descriptive statistics were calculated for all variables. Between-group comparisons for continuous variables were conducted by means of the Student's *t*-test or Kruskal–Wallis test as appropriate, while the chi-squared test was applied when comparing categorical variables. Within-group comparisons were performed using the paired Student's *t*-test. For normally distributed data, the Student's *t*-test was used for continuous variables, and Pearson chi-squared test for categorical variables. The Mann–Whitney *U* test was applied for variables that were not normally distributed.

Quality control of the registry was performed by comparing the number of required fields to the actual data entered for variables including height, weight, NPS, SNOT-22, VAS scores, SST-16 scores, ACQ, ACT, FEV_1_, FeNO, IgE, eosinophilic cell count, and treatment adherence.

## 3 Results

### 3.1 Patient disposition

**Figure 1:**
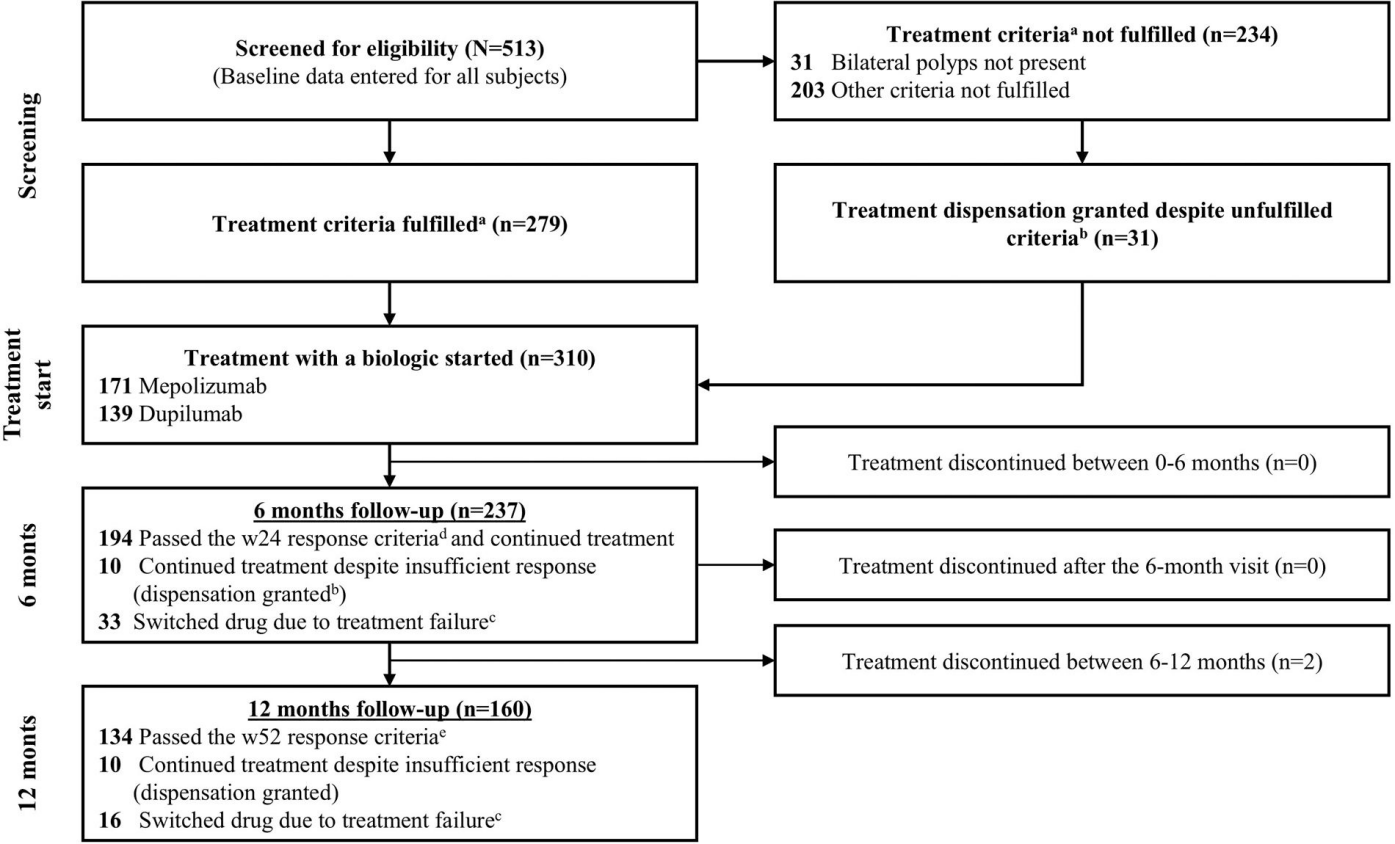
Patient disposition. ^a^Danish treatment criteria include all of the following: (1) bilateral nasal polyps, (2) ESS under general anesthesia within the last 3 years, (3) signs of type 2 inflammation, and (4) adherence to topical treatments >80%; plus three of the following: **(A)** need for systemic steroids, either two courses within a year, low dose for a period of 3 months, or a contraindication for systemic steroids, **(B)** SNOT-22 score ≥50; **(C)** anosmia, i.e., SST-16 ≤ 8; **(D)** NPS ≥5, or **(E)** asthma treated with inhaled corticosteroids. ^b^Reasons for dispensation were previous participation in a clinical trial with biologics, nasal polyps not present due to recent acute surgery (due to Pott's puffy tumor or mucocele), inability to fill out questionnaires due to intellectual disability, anxiety to surgery. ^c^The Danish treatment criteria suggest considering drug switching if the clinical response criteria are not met after six months of treatment with a biologic. ^d^Danish week-24 response criteria are at least two of the following: (1) NPS improved ≥1 point, (2) SNOT-22 improved ≥12 points, (3) ACQ improved ≥0.5 points, (4) SST-16 improved from ≤8 to >8. ^e^Danish week-52 response criteria are at least two of the following: (1) NPS improved ≥2 point, (2) SNOT-22 improved ≥12 points, (3) ACQ improved ≥0.5 points, (4) SST-16 improved from ≤8 to >8. ACQ, Asthma Control Questionnaire; ESS, endoscopic sinus surgery; NPS, Nasal Polyp Score; SNOT-22, Sinonasal Outcome Test 22; SST, Sniffin’ sticks; w24, week 24; w52, week 52.

Overall, 513 patients with CRS were referred to participating ENT departments and screened for eligibility for biologic treatment (Figure 1). Of these, 310 (60%) patients initiated treatment with biologics (mepolizumab 55%, dupilumab 45%) between November 2022 and December 2024, with 279 (54%) patients meeting the mandatory and additional criteria, and an additional 31 (6%) permitted to initiate treatment based on exceptions to the criteria. The remaining 203 patients did not fulfill the eligibility criteria and were subsequently excluded from the registry. At the data cutoff (December 2024), 237 and 160 patients had received ≥6 or ≥12 months of treatment with a biologic.

Quality control for data entry showed that at baseline, the mean proportion of missing data was 5.16%, ranging from 0.89% for NPS to 16.11% for ACQ-7. After 6 months follow-up, the mean proportion of missing data was 3.63%.

### 3.2 Baseline characteristics (phenotypes)

Baseline characteristics of the 310 patients who initiated biologic treatment are shown in Table 1. Patients had a mean age of 49.7 years, mean BMI of 27.3 kg/m^2^, and were predominantly male (66%). Furthermore, 105 (34%) were never smokers and 26 (8%) were current smokers. Overall, 204 (66%) suffered from asthma, 175 (56%) were diagnosed with allergic rhinitis, and 95 (31%) claimed a diagnosis of N-ERD. Patients with asthma had a mean (SD) ACQ-5 score of 2.1 (1.5), a mean (SD) ACT score of 17.2 (5.19) and a mean (SD) VAS asthma score of 60.2 (23.0). Overall, 187 (92%) patients with asthma had uncontrolled asthma (defined as an ACQ-5 score >1.2). Lung function measurements showed a mean (SD) FEV_1_% pred of 94.5% (18.1%), FEV_1_/FVC ratio of 69.5% (9.88%) and FeNO of 42.1 (39.0) ppb. Atopy was reported in 50% of patients.

**Table 1:** Patient demographics and baseline characteristics.

Parameter	Treated (*n* = 310)	Not treated (*n* = 203)	All patients (*N* = 513)	*P*-value^a^
Males, *n* (%)	204 (66)	134 (66)	338 (66)	0.7806^b^
BMI, mean (SD)	27.3 (5.0)	26.4 (5.4)	27.0 (5.1)	0.0454
Age, year, mean (SD)	49.7 (14.3)	49.7 (15.3)	49.7 (14.7)	0.9922
SNOT-22, mean (SD)	68.7 (18.7)	58.9 (21.1)	64.8 (20.3)	<0.0001
NPS, mean (SD)	4.8 (1.7)	4.0 (2.6)	4.6 (2.0)	0.0652
NPS, median (IQR)	5.0 (4.0, 6.0)	5.0 (2.0, 6.0)	5.0 (3.0, 6.0)	0.1549^c^
NCS, mean (SD)	2.4 (0.7)	2.3 (0.7)	2.3 (0.8)	0.6781
VAS CRS, mean (SD)	84.1 (16.2)	78.7 (21.6)	82.0 (18.6)	0.0054
VAS Smell, mean (SD)	91.3 (19.4)	78.1 (29.5)	86.1 (24.7)	<0.0001
SST-16 score, mean (SD)	4.8 (3.1)	7.8 (4.6)	6.0 (4.0)	<0.0001
VAS N-ERD, mean (SD)	82.9 (28.6)	75.3 (30.7)	80.5 (29.4)	0.1668
Blood EOS, ×10^6^ /L, mean (SD)	0.7 (4.3)	0.4 (0.4)	0.6 (3.5)	0.2278
Blood IgE kU/L, mean (SD)	233.1 (437.4)	230.4 (530.6)	232.1 (473.6)	0.9539
ACQ-5, mean (SD)	2.1 (1.5)	1.7 (1.4)	2.0 (1.5)	0.0003
ACQ-7, mean (SD)	1.9 (1.2)	1.5 (1.1)	1.7 (1.2)	0.0012
ACT, mean (SD)	17.2 (5.2)	18.6 (5.1)	17.7 (5.2)	<0.01
VAS Asthma, mean (SD)	60.2 (23.0)	58.5 (24.1)	59.6 (23.4)	0.5079
FEV_1_%pred, mean (SD)	94.5 (18.1)	92.7 (22.2)	93.8 (19.8)	0.3439
FVC %pred, mean (SD)	107.4 (20.3)	102.1 (22.0)	105.3 (21.2)	0.0075
FeNO, ppb, mean (SD)	42.1 (39.0)	37.8 (29.0)	40.4 (35.4)	0.1609
Atopy^d^, *n* (%)	50 (6)	60 (6)	50 (6)	0.2872

Demographics and baseline characteristics for the overall population and for the patients treated with biologics versus not treated with biologics.

VAS is a subjective score of severity where 0 = best and 100 = worst.

ACQ, Asthma Control Questionnaire; BMI, body mass index; CRS, chronic rhinosinusitis symptoms; EOS, eosinophil cell count; FeNO, fractional exhaled nitric oxide; FEV_1_, forced expiratory volume in 1 s; FVC, forced vital capacity; IQR, interquartile range; NCS, Nasal Congestion Score; NPS, Nasal Polyp Score; N-ERD, non-steroidal anti-inflammatory drug-exacerbated respiratory disease; SD, standard deviation; SNOT-22, Sinonasal Outcome Test 22; SST, Sniffin’ sticks; VAS, visual analog scale.

a P-values are for the treated vs. non-treated patient groups and are based on the Student's *t*-test unless otherwise indicated.

b Chi-square test.

c Kruska-Wallis test.

d Indicating either a positive skin prick test or ≥1 positive specific IgE to standard aeroallergens.

The mean (SD) number of previous ESS was 2.4 (1.8) and the mean (SD) NPS was 4.8 (1.7). Mean (SD) patient-reported outcomes were 68.7 (18.7) for SNOT-22 score, 84.1 (16.2) for VAS CRS, 91.3 (19.4) for smell dysfunction VAS, 82.9 (28.6) for VAS N-ERD symptoms, and 4.8 (3.1) out of 16 for SST-16 score. Median Nasal Congestion Score (NCS) was 3 (range 1–3). Overall, 95% of patients had a SNOT-22 score >40% and 89% had an SST-16 score ≤8. The mean (SD) blood eosinophilic cell count was 0.7 × 10^6^ /L (4.3), and eosinophilic cell counts in polyp tissue were low in 16% of patients, moderate in 37% and high in 46%. All patients met the mandatory criteria of T2 inflammation.

Patients who met the criteria for biologic treatment had significantly greater disease severity (SNOT-22, VAS CRS and Smell) and more severe asthma (ACQ-5, ACQ-7, ACT) than those who did not meet the criteria (Table 1).

### 3.3 Follow-up results

After six months of treatment, 194 of 237 patients (81%) met the continuation criteria (Figure 1), with 58 meeting two of the four criteria, 73 meeting three, and 63 meeting all four. Among the 43 patients who did not meet the threshold, 38 met only one criterion and five did not meet any. Of these 43 patients, 33 patients were switched to the alternative biologic while 10 continued treatment despite not meeting the criteria due to specific medical considerations. After 12 months, 134 of 160 patients (84%) met the continuation criteria, with 34 meeting two criteria, 48 meeting three, and 52 meeting all four. Among the 26 patients who did not meet the continuation threshold, 17 met only one criterion and nine did not meet any. Of these 26 patients, 16 were switched to the alternative biologic while 10 continued treatment due to medical considerations. No patients required either SCS or re-operation during the 12-month period.

Over the first 6 months of treatment, statistically significant improvements in multiple clinical outcomes were observed, including decreases in SNOT-22 score (68 to 29), VAS CRS (84 to 40), ACQ-5 (2.1 to 0.9) and FeNO (40.4 to 27.3) and increases in SST-16 smell score (4.7 to 9.1) (Table 2). The proportions of patients meeting the individual continuation criteria are shown in Table 3. A SNOT-22 reduction ≥12 points was reported by 193 patients (81%), a reduction in NPS by ≥1 point was reported by 167 patients (71%) and a reduction in ACQ-5 by ≥0.5 was reported by 133 patients (56%). The proportion of patients with an SST-16 smell score of 9–16 indicating normosmia increased from 11% to 51%.

**Table 2:** Clinical and patient-reported outcomes at baseline and 6-month follow-up for patients with ≥6 months of treatment.

Parameter	Baseline (*n* = 237)	Month 6 (*n* = 237)	*P*-value^a^
SNOT-22, mean (SD)	68.1 (18.4)	29.3 (21.4)	<0.0001
NPS, mean (SD)	4.7 (1.7)	2.9 (2.2)	<0.0001
NPS, median (IQR)	5.0 (3.0, 6.0)	2.0 (1.0, 4.0)	<0.0001^b^
NCS, mean (SD)	2.4 (0.7)	1.2 (0.8)	<0.0001
VAS CRS, mean (SD)	84.4 (15.1)	39.6 (28.1)	<0.0001
VAS Smell, mean (SD)	91.0 (19.9)	52.8 (35.7)	<0.0001
SST-16, mean (SD)	4.7 (3.0)	9.1 (4.7)	<0.0001
VAS N-ERD, mean (SD)	81.4 (30.7)	48.6 (27.0)	<0.0001
Blood EOS, ×10^6^ /L, mean (SD)	0.8 (5.0)	0.3 (0.4)	0.1505
ACQ-5, mean (SD)	2.1 (1.5)	0.9 (1.0)	<0.0001
ACQ-7, mean (SD)	1.9 (1.2)	1.0 (0.8)	<0.0001
ACT, mean (SD)	17.3 (5.2)	21.4 (4.1)	<0.001
VAS Asthma, mean (SD)	60.5 (22.7)	31.0 (22.3)	<0.0001
FEV_1_%pred, mean (SD)	94.7 (18.3)	97.6 (19.2)	<0.0001
FVC %pred, mean (SD)	’ 106.4 (16.9)	109.0 (24.0)	0.0102
FeNO, ppb, mean (SD)	40.4 (37.1)	27.3 (23.0)	<0.0001

VAS is a subjective score of severity where 0 = best and 100 = worst.

ACQ, asthma control questionnaire; CRS, chronic rhinosinusitis symptoms; EOS, eosinophil cell count; FeNO, fractional exhaled nitric oxide; FEV_1_, forced expiratory volume in 1 s; FVC, forced vital capacity; IQR, interquartile range; NPS, Nasal Polyp Score; N-ERD, non-steroidal anti-inflammatory drug-exacerbated respiratory disease; SD, standard deviation; SNOT-22, Sinonasal Outcome Test 22; SST, Sniffin’ sticks; VAS, visual analog scale.

a *P*-values are for the baseline versus 6-month data and are based on the Student's *t*-test unless otherwise indicated.

b Kruska-Wallis test.

**Table 3:** Proportions of patients achieving continuation criteria after 6 and 12 months of treatment.

Criteria for continuation	Month 6 (*n* = 237)	Month 12 (*n* = 160)
NPS reduction of ≥1 point, *n* (%)	167 (71%)	
NPS reduction of ≥2 points, *n* (%)		104 (65%)
SNOT-22 reduction ≥12, *n* (%)	193 (81%)	130 (81%)
SST-16 score 9–16, *n* (%)	120 (51%)	95 (59%)
ACQ-5 reduction of 0.5, *n* (%)	133 (56%)	101 (63%)

ACQ, asthma control questionnaire; NPS, nasal polyp score; SNOT-22, sinonasal outcome test 22; SST, sniffin’ sticks.

**Figure 2:**
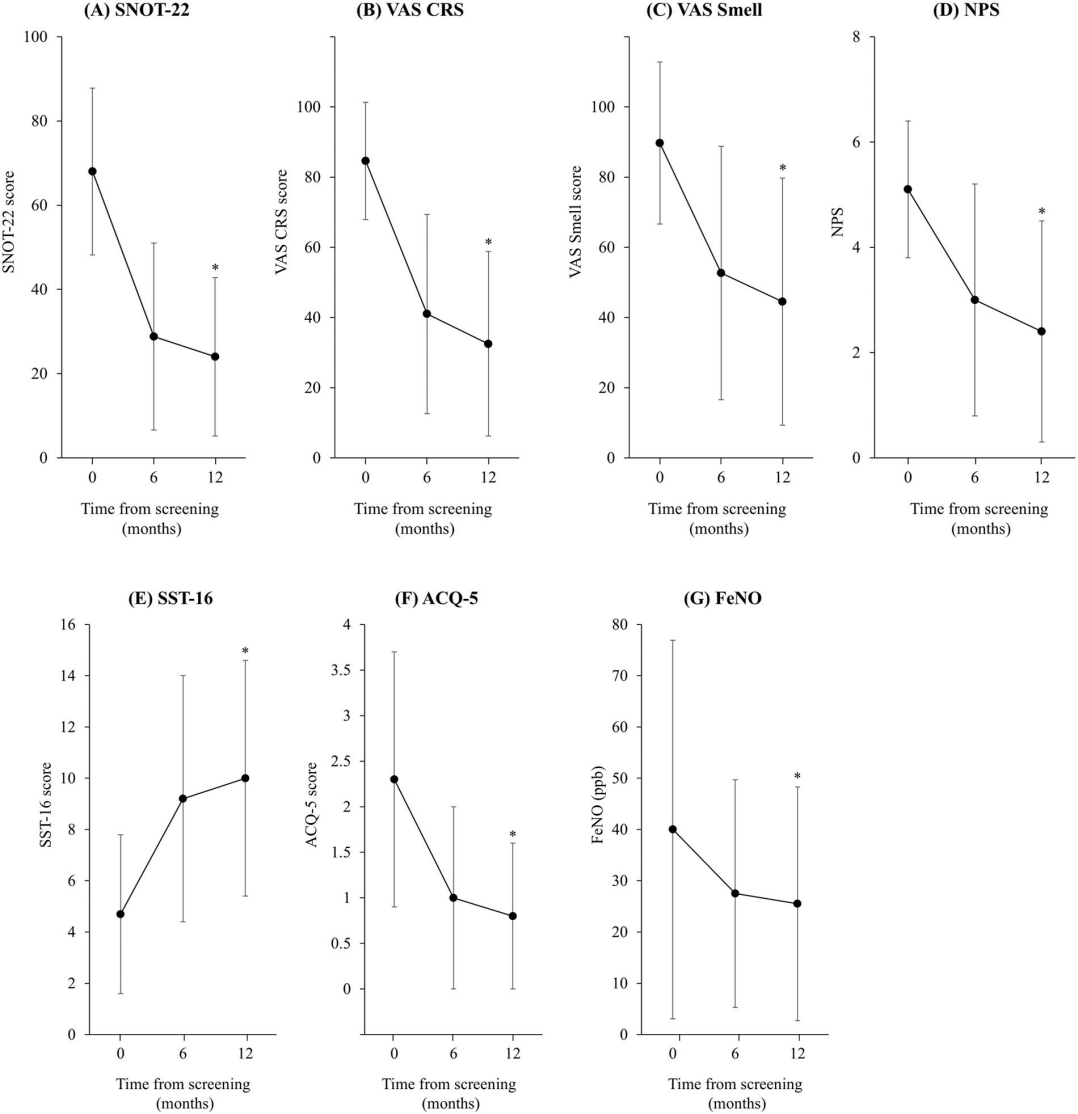
Change from baseline to Month 12 for **(A)** SNOT-22 score, **(B)** VAS CRS, **(C)** VAS smell, **(D)** NPS, **(E)** SST-16 score, **(F)** ACQ-5 score and **(G)** FeNO. Data shown are mean (SD). VAS is a subjective score of severity where 0 = best and 100 = worst. *p < 0.0001. *P*-values are for the baseline versus 12-month data and are based on the Student's *t*-test. ACQ, Asthma Control Questionnaire; CRS, chronic rhinosinusitis symptoms; FeNO, fractional exhaled nitric oxide; NPS, Nasal Polyp Score; SD, standard deviation; SNOT-22, Sinonasal Outcome Test 22; SST, Sniffin’ sticks; VAS, visual analog scale.

Among patients with ≥12 months of treatment (*n* = 160), a reduction in disease burden (both upper and lower airway involvement) was observed over the 12 months follow-up (Figure 2). Overall, SNOT-22 reduced from 68 at baseline to 29 at month 6 and 24 at month 12, NPS reduced from 5.1 to 3.0 and 2.4, the SST-16 smell score increased from 4.7 to 9.2 and 10.0, ACQ-5 reduced from 2.3 to 1.0 and 0.8, and FeNO normalized from 40.0 to 27.5 and 25.5. Statistically significant improvements from baseline to month 12 were observed for most parameters (Table 4).

**Table 4:** Clinical and patient-reported outcomes at baseline, 6-month follow-up and 12-month follow-up for patients with ≥12 months of treatment.

Parameter	Baseline (*n* = 160)	Month 6 (*n* = 160)	Month 12 (*n* = 160)	*P*-value baseline vs. month 12^a^	*P*-value baseline vs. month 6 vs. month 12^b^
Sinonasal outcomes					
SNOT-22, mean (SD)	68.0 (19.8)	28.8 (22.2)	24.0 (18.8)	<0.0001	0.0010
NPS, mean (SD)	5.1 (1.3)	3.0 (2.2)	2.4 (2.1)	<0.0001	0.0612
NPS, median (IQR)	5.0 (3.0, 6.0)	2.0 (1.0, 4.0)	2.0 (1.0. 4.0)	<0.0001^c^	<0.0001^c^
VAS CRS, mean (SD)	84.6 (16.7)	41.0 (28.4)	32.5 (26.3)	<0.0001	0.0016
NCS, mean (SD)	2.5 (0.7)	1.2 (0.8)	1.0 (0.8)	<0.0001	0.0109
VAS Smell, mean (SD)	89.7 (23.1)	52.7 (36.1)	44.5 (35.2)	<0.0001	0.0010
SST-16 score, mean (SD)	4.7 (3.1)	9.2 (4.8)	10.0 (4.6)	<0.0001	0.0005
VAS N-ERD, mean (SD)	78.9 (30.7)	46.1 (30.0)	53.4 (32.6)	0.0017	0.5132
Bronchial outcomes					
ACQ-5, mean (SD)	2.3 (1.4)	1.0 (1.0)	0.8 (0.8)	<0.0001	0.0014
ACQ-7, mean (SD)	2.0 (1.2)	1.1 (0.9)	0.9 (0.7)	<0.0001	0.0072
ACT, mean (SD)	16.9 (5.1)	22.3 (4.1)	22.0 (3.4)	<0.001	0.055
VAS Asthma, mean (SD)	60.9 (22.5)	32.6 (21.3)	26.0 (20.0)	<0.0001	<0.0001
FEV_1_%pred, mean (SD)	93.8 (19.0)	97.7 (18.7)	96.2 (17.7)	0.0101	0.8488
FVC %pred, mean (SD)	106.4 (17.6)	109.8 (25.8)	110.0 (8.6)	0.2200	0.2335
Type 2 inflammation					
Blood EOS, ×10^6^ /L, mean (SD)	1.0 (6.2)	0.2 (4.8)	0.3 (0.4)	0.2008	0.0030
FeNO, ppb, mean (SD)	40.0 (36.9)	27.5 (22.2)	25.5 (22.8)	<0.0001	0.2139

VAS is a subjective score of severity where 0 = best and 100 = worst.

ACQ, Asthma Control Questionnaire; CRS, chronic rhinosinusitis symptoms; EOS, eosinophil cell count; FeNO, fractional exhaled nitric oxide; FEV_1_, forced expiratory volume in 1 s; FVC, forced vital capacity; IQR, interquartile range; NPS, Nasal Polyp Score; N-ERD, non-steroidal anti-inflammatory drug-exacerbated respiratory disease; SD, standard deviation; SNOT-22, Sinonasal Outcome Test 22; SST, Sniffin’ sticks; VAS, visual analog scale.

a *P*-values are for the baseline versus 12-month data and are based on the Student's *t*-test unless otherwise indicated.

b *P*-values are for the baseline versus 6-month versus 12-month data and are based on the Student's *t*-test unless otherwise indicated.

c Kruska-Wallis test.

No serious adverse reactions were reported for dupilumab or mepolizumab by the 160 patients who received treatment for ≥12 months. Twenty-four patients reported milder side effects.

## 4 Discussion

The establishment of the Global Airways registry represents a significant advance in the management of CRSwNP and comorbid asthma in Denmark, enabling systematic, longitudinal assessment of both upper and lower airway disease in real-world clinical practice. The low proportion of missing data enhances the reliability of these findings, and the observed improvements in both sinonasal and asthma outcomes support the effectiveness of biologic therapy in this well-characterized patient population.

The Global Airways registry is among the first to collect real-world data through a nationwide database within the public health system and supports ENT specialists as they transition from a primary focus on surgery to the use of biologics in CRSwNP management. The variables collected in Global Airways align with a set of clinically-relevant variables that were identified by Delphi consensus for inclusion in the international INVENT registry that aims to consolidate data on biologic use in CRSwNP (32). At the start, many ENT specialists were more familiar with surgical approaches and were less aware of comorbid asthma management and T2 inflammation. Over time, however, the registry has facilitated a broader understanding of both asthma and T2 mechanisms, serving as an important educational tool. Its nationwide, multicenter design captures all Danish patients receiving biologics for CRSwNP, with systematic assessment of asthma and other T2 comorbidities. Few other studies have measured FeNO systematically in patients with CRSwNP despite its value as a standardized measure of T2 inflammation in asthma patients. Combined with Denmark's universal healthcare system, this approach ensures equitable access and high-quality longitudinal data. Compared with other registries, which may be single-center, retrospective, or lack integrated asthma work-up (24, 25, 33–37), this approach provides a comprehensive real-world view of biologic efficacy. Our registry also provides insights into evolving treatment practices. The actual number of treated patients was lower than expected (8) and SCS use was minimal in recent years, reflecting a shift toward steroid-sparing strategies as cumulative side effects became better documented (38, 39).

Over 12 months of treatment with biologics, patients in our cohort achieved sustained control of nasal symptoms, normalization of self-reported disease burden, improved smell function and normalization of FeNO. Patients also experienced a substantial reduction in asthma burden, which exceeded the MCID of 0.5 and outperformed other real-world studies (23, 40) and a clinical trial (21). At six months, more than half of patients had an excellent response to biologics per EPOS/EUFOREA criteria (28), which was higher than in a previous observational study (41), although the combined proportions of excellent and moderate responders in the two studies were similar. Sinonasal outcomes in our cohort and other real-world cohorts (23, 40, 42) were generally superior to those from phase 3 trials of dupilumab and mepolizumab (18, 43), as well as for other biologics (20–22).

The high baseline disease severity in our cohort is a key consideration. Danish eligibility criteria for biologic therapy are stricter than EPOS/EUFOREA 2023 criteria (28), and as a result all included patients had bilateral NPs despite prior ESS. This suggests that the patients had rapidly developed new polyps since ESS, which is indicative of aggressive disease, and potentially more responsive disease. Almost all patients had severe symptom burden, frequent smell dysfunction, and a high prevalence of comorbid asthma. Inflammatory profiling confirmed a T2 inflammatory phenotype, with elevated blood eosinophils, high IgE, and, where available, biopsy-confirmed eosinophilia. Blood eosinophilia levels were similar to another real-world study (40) but higher than a phase 3 study (21). Our cohort also differed from the real-world study by De Corso et al. (24), where 28% of patients had never undergone ESS, though, that was similar to one clinical trial (20).

Two-thirds of our cohort met the GINA asthma criteria (44), with uncontrolled asthma at baseline (ACQ >1.2) reported by 92% of asthma patients. The mean ACT value of 17 is worse than previously reported in a Dutch real-world cohort (23). Previous studies have found that patients with CRSwNP generally have mild to moderate asthma (44, 45), with the most severe cases in those needing repeated surgery (46). While a ‘doctor's diagnosis of asthma’ can be used in large cohort studies without skewness (47), it should not be applied in clinical studies due to difficulties in accurately diagnosing asthma (48). Near-normal FEV_1_ in moderate asthma combined with low frequency of smoking in our cohort support a more accurate asthma diagnosis and treatment response.

Comparing outcomes between our study and other real-world studies or phase 3 clinical trials is challenging due to differences in inclusion criteria and treatment duration. Although it is possible that the high baseline disease severity in our cohort contributed to the relatively large treatment response, chronic damage resulting from long-standing inflammation and delayed access to disease-modifying treatments could conversely limit improvement. The improvements observed in asthma control in our study could also reflect the close ENT-pulmonology collaboration in Denmark, enabling ICS adjustment alongside biologic therapy. Another possible reason for the outcomes we observed is that the strict Danish response criteria (requiring a ≥2-point NPS improvement after 12 months vs. ≥1 point in EPOS/EUFOREA) prompted biologic switching in some cases. This approach is also facilitated by the fact that Denmark has two reimbursed biologics available. However, the clinical benefits of switching between biologics are unknown and data remain limited.

Limitations of our study include site size variation, with smaller rural sites contributing fewer patients than university hospitals, despite the strengths of the multicenter design.

We are currently conducting a real-world, randomized, head-to-head, multicenter comparison between dupilumab and mepolizumab (TORNADO; EU Clinical Trial ID: 2022-50-22-50-14-00, clinicaltrials.org ID: NCT05942222), with results expected in early 2026. The merging of real-world data with similar inclusion criteria followed by uniform analysis could further enhance understanding of biologic treatment outcomes in CRSwNP.

In conclusion, the Global Airways registry provides a robust foundation for ongoing research and clinical practice in the treatment of CRSwNP and comorbid asthma. Our findings demonstrate the sustained effectiveness of biologic therapy in patients with recalcitrant CRSwNP and T2 inflammation, as well as in associated comorbidities such as asthma. These insights will be instrumental in defining phenotypes of CRS and their associated comorbidities, supporting the development of more targeted and effective treatments for patients worldwide.

## Data Availability

The raw data supporting the conclusions of this article will be made available by the authors, without undue reservation.
