# Supplementary material for: Global airways: a Danish nationwide real-life registry of biologic therapy for chronic rhinosinusitis with nasal polyps
Source: Front Allergy. 2026 Jan 13;6:1735943. doi: 10.3389/falgy.2025.1735943 (PMC12834761; doi:10.3389/falgy.2025.1735943)
Supplement: Supplementary file 1 [file Datasheet1.docx]

**Supplementary Appendix 1. Variables collected in the Global Airways registry**

1. Date of birth
2. Height
3. Weight
4. Tobacco consumption
5. Adherence to intranasal corticosteroids, inhaled corticosteroids, saline irrigation
6. Use of systemic corticosteroids
7. Fractional exhaled nitric oxide
8. Forced expiratory volume in 1 second
9. Forced vital capacity
10. Sniffin’ Sticks-16
11. Blood eosinophilic count
12. IgE kU/L
13. Allergy testing (specific IgE or skin prick test)
14. Questionnaires: Standard Tests for Asthma, Allergic Rhinitis, and Chronic Rhinosinusitis (STARR15), sinonasal outcome test (SNOT-22), Asthma Control Questionnaire (ACQ), Asthma Control Test (ACT), Medication Adherence Report Scale (MARS5) and Eustachian Tube Dysfunction Questionnaire (ETDQ-5)
15. Nasal Congestion Score
16. Visual Analog Scales for chronic rhinosinusitis symptoms, Allergy, Smell, Asthma, and VAS NSAID-exacerbated respiratory disease symptoms
17. Symptoms of chronic rhinosinusitis (nasal obstruction, nasal discharge, facial pain and smell dysfunction)
18. Symptoms of asthma (shortness of breath at rest or exercise, cough, wheezy, chest tightness or nighttime symptoms)
19. Asthma was diagnosed based on symptoms and a bronchial challenges test either current or a history of a test
20. Symptoms of allergic rhinitis (runny nose, itchy nose and eyes, sneezy, seasonal variation)
21. CT sinuses and calculation of Lund-Mackay score (performed by the program)
22. Rhinoscopy with Nasal Polyp Score defined (0-4 or each side)
23. History of endoscopic sinus surgery
24. Number of polypectomies and number of sinus surgeries performed
25. Date of the last sinus surgery
26. Nasal polyp pathology with scoring of mild/moderate/severe eosinophilic infiltration
27. Conclusion and treatment decision

**Supplementary Appendix 2. Registry procedures**

Data entry in the registry can be performed in three blocks: one by trained airway nurses, one by patients with a QR code and, one by doctors. Data entry does not have to be completed in a single day, and examinations can be separated into two or more parts, each assigned a date. The research staff assign an ID number to each patients, with a suffix for the geographic region of examination. Staff then initiate the systematic assessment, including date of birth, height, weight, tobacco consumption, adherence to intranasal corticosteroids (INCS), inhaled corticosteroids (ICS) and saline irrigation. The program calculates the percentage of adherence automatically.

The use of systemic steroids (SCS) over two years (or five years if the patient can recall), as well as the total sum of SCS, injections and tablets, are calculated by the program. Moreover, measurements of fractional exhaled nitric oxide, forced expiratory volume in 1 second (FEV_1_) and forced vital capacity (FVC) are performed, with % predicted values for FEV_1_ and FVC calculated based on the patient’s age, height and weight. Nurses perform the Sniffin’ Sticks 16 test, and smell scores are automatically calculated when entered directly into the program. Finally, nurses or other research staff enter the eosinophilic count, IgE (kU/L) and allergy testing results (specific IgE or skin prick test).

The following questionnaires are completed: Standard Tests for Asthma, Allergic Rhinitis, and Chronic Rhinosinusitis (STARR15), sinonasal outcome test (SNOT-22), Asthma Control Questionnaire (ACQ), Asthma Control Test (ACT), Medication Adherence Report Scale (MARS5) and Eustachian Tube Dysfunction Questionnaire (ETDQ-5; only since July 2024), as well as Nasal Congestion Score (NCS) and Visual Analog scales for chronic rhinosinusitis symptoms, Allergy, Smell, Asthma and N-ERD symptoms.

The medical history and examination include symptoms of chronic rhinosinusitis (CRS; nasal obstruction, nasal discharge, facial pain and smell dysfunction), defining chronic CRS disease > 12 weeks and symptoms of asthma (shortness of breath at rest or exercise, coughing, wheezing, chest tightness or nighttime symptoms). Asthma is diagnosed based on symptoms and a bronchial challenge test, either current or a history of a test, symptoms of allergic rhinitis (runny nose, itchy nose and eyes, sneezing, seasonal variation), CT sinuses, calculation of Lund McKay score (LMK) by the program, rhinoscopy with Nasal Polyp Score (NPS) defined (0–4 for each side), history of ESS, number of polypectomies, date of the last ESS, and nasal polyp pathology (historic) with scoring of mild/moderate/severe infiltration of eosinophilic cells. Finally, the systematic assessment terminates with a conclusion and a treatment decision. The program produces an outputs containing all relevant total scores and values needed to make a final decision. Prior to medical evaluation, all patients must complete most of the examinations, as many of the test values are needed to make decisions concerning local and systemic treatment of the upper and lower airways.

Prior to the initiation of the registry, local meetings at all sites were performed to educate the staff on Global Airways diseases, systematic assessments, local treatment with INCS and ICS, and the workflow of the registry, which has been developed as a clinical support tool. The same examinations are performed every six months. A shorter visit is performed four months after initiation of biologic therapy, with a focus on systemic or local side effects. A yearly report is prepared, after which quality control can be performed. Data revision at the nine sites has been requested. In addition, a midterm report has been prepared with the number of patients included in the registry and the treatment response, which have been explained at a face-to-face meeting with both research staff and doctors present.
